# Design and Experimental Demonstration of Pyramidal SERS Substrates with High-Throughput Fabrication Method

**DOI:** 10.1021/acsomega.4c11431

**Published:** 2025-05-24

**Authors:** Fabiano C. Santana, Yuri H Isayama, Rafael S. Gonçalves, Felipe M. F. Teixeira, Jhonattan C Ramirez, Wagner N. Rodrigues

**Affiliations:** † Departamento de Física, Instituto de Ciências Exatas, 28114 Universidade Federal de Minas Gerais, Belo Horizonte, MG 31270-901, Brazil; ‡ Departmento de Engenharia Eletrônica, Escola de Engenharia, Universidade Federal de Minas Gerais, Belo Horizonte, MG 31270-901, Brazil; § Programa de Pós-Graduação em Engenharia Elétrica, Escola de Engenharia, 8101 Universidade Federal de Minas Gerais, Belo Horizonte, MG 31270-901, Brazil; ∥ Centro de Microscopia, Universidade Federal de Minas Gerais, Belo Horizonte, MG 31270-901, Brazil

## Abstract

Surface-enhanced Raman spectroscopy (SERS) is a technique based on the Raman scattering effect that provides rapid, label-free identification capabilities with very high sensitivity and specificity down to the molecular level. Due to the nature of the applications, not only are sensitivity and specificity important, but also the reproducibility of substrates is a key aspect. In this study, a design methodology for arrays of pyramid-shaped gold nanostructures is presented along with the development of a fabrication process that enables high-throughput substrate production. Using simulations of the 3D finite element method, SERS substrates with theoretical enhancement factors on the order of 10^8^ have been obtained. SERS experiments with the fabricated structures resulted in an enhancement of approximately 1500% compared to flat gold substrates. The thorough investigation of the structure, allied with a novel fabrication process that eliminates the need for complex and time-consuming lithography steps for each individual substrate, paves the way for an efficient method to produce high-quality SERS substrates.

## Graphical abstract

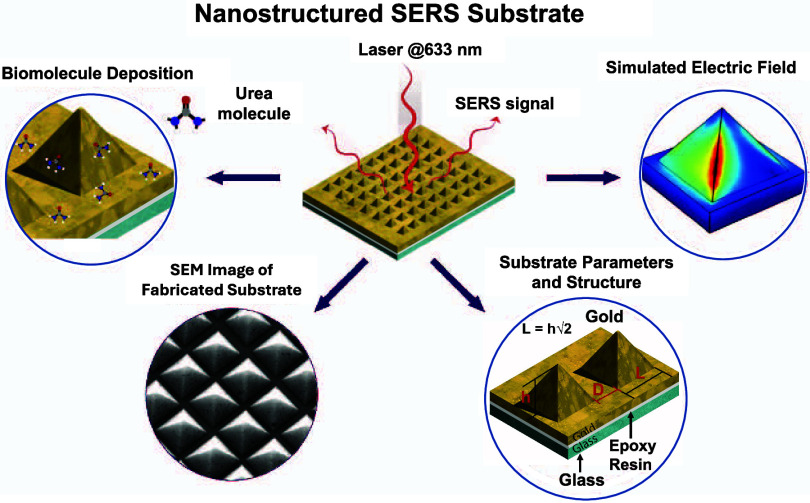

## Introduction

Raman spectroscopy is a cutting-edge analytical technique that has had a major impact in a wide variety of fields, among which one can cite: chemistry and biology,^1–5^ materials science,^6,7^ pharmaceutical and medical,^8,9^ and food industries.^10,11^ Its ability to provide rapid and label-free identification with specificity down to the molecular level has made it a powerful asset in nanoscale characterization processes.^12,13^ However, the main limitation associated with Raman scattering is that it is inherently a weak intensity effect.^14,15^

In order to overcome this drawback, extensive research has been conducted over the last five decades with the objective of increasing the sensitivity of this type of spectroscopy, leading to the field of Surface-enhanced Raman spectroscopy (SERS). The SERS technique strongly benefits from a phenomenon called surface plasmon resonance (which can be spatially localized or not), which occurs when a metal structure is illuminated by an incident light source and surface plasmons are excited, leading to the generation of high-intensity electromagnetic (EM) fields in the region close to the metal surfaces.^15,16^ As Raman scattering is highly affected by field intensity, the effect can be greatly enhanced using adequately structured metal substrates.

There have been many different approaches to the development of SERS substrates, which can mainly be separated in the following ways: forming micro- and nanostructures on the surface (wrinkles, pyramids, blocks, disks, etc.);^1,17–25^ and depositing or growing nanoparticles on the substrates.^14,18,25–33^

Although the use of deposited or grown nanoparticles provides very good Raman signal enhancement, one important aspect of a SERS substrate is its structural reproducibility, which is one of the advantages provided by nanofabrication processes. It has been reported that substrates can be obtained through simple, reproducible fabrication procedures.^1,20,34^ Furthermore, since the shape of micro and nanostructures has a large impact on the intensity of generated EM fields, using consolidated nanofabrication techniques combined with a thorough and careful design step may produce more predictable and controlled results.^1,20,24,34^

In this work, we present the numerical design and experimental demonstration of a SERS substrate comprising a matrix of gold pyramids on a gold layer. The pyramidal nanostructure shape in this case is defined by the well-known anisotropic etching of (100) oriented silicon in KOH solution, which results in pyramidal pits exposing (111) crystal planes.^35–38^ The problem symmetry in this case cannot be correctly simplified to a two-dimensional analysis, thus the design step was developed using 3D-Finite Element Method (3D-FEM) simulations, where pyramid dimensions (edge length and spacing) were the design parameters, as well as the polarization of the incident light. The design quality was evaluated on the basis of the electric field enhancement on the surface of the structured substrate compared to that of a flat gold substrate. Due to the reproducibility requirement, a fabrication method was developed to allow serial production of SERS substrates with small geometry variation, by means of a silicon mold that can be used as a base for the production of the pyramid matrix substrate. The mold may be reused, eliminating the need for lithography in every substrate production. The resulting SERS substrate showed roughly a 1500% improvement in Raman response compared to a flat gold substrate, demonstrating a promising approach to large-scale production of SERS substrates.

## SERS Substrate Design

Raman scattering is typically a weak process for the great majority of molecules, as Raman scattering cross sections are generally very small. Surface-enhanced Raman spectroscopy (SERS) employs plasmonic nanostructures in a way that, upon light excitation, surface plasmon resonances arise within the substrate.^39^ These resonances generate electromagnetic (EM) hotspots near the metallic surfaces, which, in turn, result in an amplification of radiation emission.

An important parameter for quantifying the efficiency of the SERS substrate is the enhancement factor (EF), which can be approximated by EF ≈ |*E*(ω)|^4^/|*E*_0_|^4^, where |*E*(ω)| corresponds to the electric field amplitude resulting from the excitation of plasmons in the SERS substrate (at a frequency ω) and *E*_0_ is the incident electric field amplitude.^40^ Thus, there are two requisites for the construction of an efficient SERS substrate: first, the EM fields near the metallic surface, where analytes will be located, must be as intense as possible; second, since the molecules of interest have to be exposed to these intense fields, the energy density also has to be high. If the substrate is solely structured to increase the EM field intensity without taking energy density into account, it could happen that the molecules under analysis fail to be exposed to EM radiation, and therefore, Raman scattering will not be enhanced.

In order to take into account the spatial distribution of hotspots, an adaptation of the enhancement factor (EF′) was considered in the substrate design

1 $$EF^{\prime }=\frac{\int_{S}|E\left(\omega \right){|}^{4}\;dS}{\int_{A}|E_{0}{|}^{4}\;dA}$$

where *S* denotes the surface comprised by the SERS substrate and *A* refers to the surface of a flat gold substrate with the same footprint area. Here, the evaluation of the surface integral of EF considers the context of molecule detection: if an analyte does not fall within the area subject to an enhanced field, Raman scattering will not be enhanced. Therefore, looking solely at the maximum field enhancement in a single point might produce the false idea that a certain substrate configuration has better performance than one with less intense electric fields, but with a more distributed spatial profile.

Another important factor when designing an SERS substrate is the need for large quantities of substrates because of the very nature of SERS applications. The fabrication process of the substrate must be inexpensive and reproducible. Hence, for its simple fabrication process, the square-based pyramid shape (all eight edges with the same length) that results from anisotropic etching of (100)-oriented silicon was selected as the base structure for the design. The structured substrate is composed of a matrix of gold pyramids, as depicted in Figure 1, which presents two degrees of freedom in terms of design: edge size and separation between pyramids. The goal is to find a combination of pyramid spacing and edge length that maximizes the energy in the surface of the pyramids (increasing plasmonic response), while also maintaining a high energy density within the unit cell that repeats throughout the pyramid matrix.

**Figure 1:**
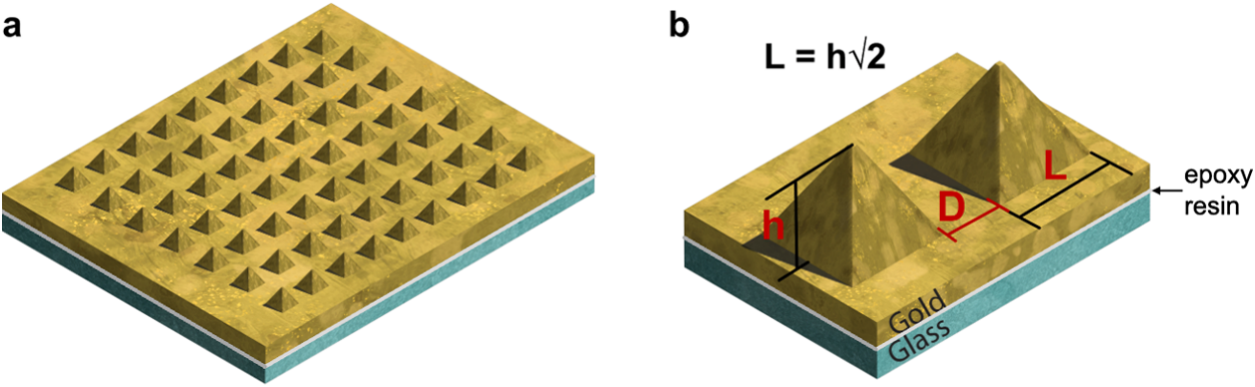
Schematic of the fabricated SERS substrate: (a) array of square-based nanometric gold pyramids on a gold film fixed to a glass slide through an epoxy resin layer; (b) parameters of the structure - *L* is the length of an edge of the pyramid basis, *D* is the distance between adjacent pyramids, and *h* is the height.

Simulations were performed using the three-dimensional Finite Element Method (3D-FEM), which, due to symmetry, used a unit cell that repeats in the *x* and *y* directions. The size of the domain varied with the dimensions of the pyramids and the spacing:

x×y×z=(L+D)×(L+D)×(2L)

where *L* is the length of the edge of the regular square pyramid and the spacing of the pyramid *D* is the distance between the edges of two adjacent pyramids. The boundary conditions were configured as follows: Perfect Electric Conductor beneath the substrate; Periodic Boundary Conditions on the four lateral sides of the unit cell; and a port input on the top of the cell, where EM field excitation took place in the form of a plane wave, with variable elevation and azimuth angle. The material surrounding the pyramids and above the substrate was considered air, and both the pyramids and the substrate were made of gold. As the Raman spectroscopy equipment used in this study presented a confocal geometry with both the light source and detection in the same direction, initial simulations were performed considering a zero-degree elevation angle (normal incidence with respect to the substrate surface). The chosen wavelength of operation was 633 nm due to the available characterization equipment, but the same engineering method should be able to be applied to different wavelengths or substrate geometries. For each substrate configuration (that is, *D* and *L* values), the adapted enhancement factor, EF′, given by eq 1 was calculated and used as a metric to compare the different design geometries.

### Simulation Results

The first step was to investigate how the electric field distribution in a flat sheet made of gold would be affected by the presence of the pyramidal geometry. Figure 2 depicts the intensity of the electric field on a gold flat surface (left) and on the surface of gold pyramids (right), assuming the normal incidence of a plane wave (with θ = 0° and ϕ = 0°) and pyramids with dimensions *L* = 400 nm (edge) and *D* = 100 nm (spacing). For the gold flat surface, most of the incident light is reflected, and because there is a continuous incident plane wave, a stationary wave is formed above the gold surface. The electric field present on the gold surface indicates the excitation of surface plasmons, but as is known from the SPR theory, the angle of incidence of the plane wave plays an important role in the efficiency of plasmon excitation.^41,42^ As the incidence angle is zero (normal incidence), this efficiency is small and almost all incident light is simply reflected. However, on the right side of Figure 2, it is clear that the pyramidal shape significantly alters the behavior of the electric field distribution. Even though there is still the formation of a stationary wave, electric fields are much more concentrated in the region near to the surface of the pyramid. In addition, with an azimuth angle of 0°, one can observe that the plasmons are excited and that the field **E** is concentrated along the lateral edges of the pyramid. In addition, there is a considerable intensity of electric field in the region close to the lateral faces of the pyramid, where molecules may be deposited, and thus sensed.

**Figure 2:**
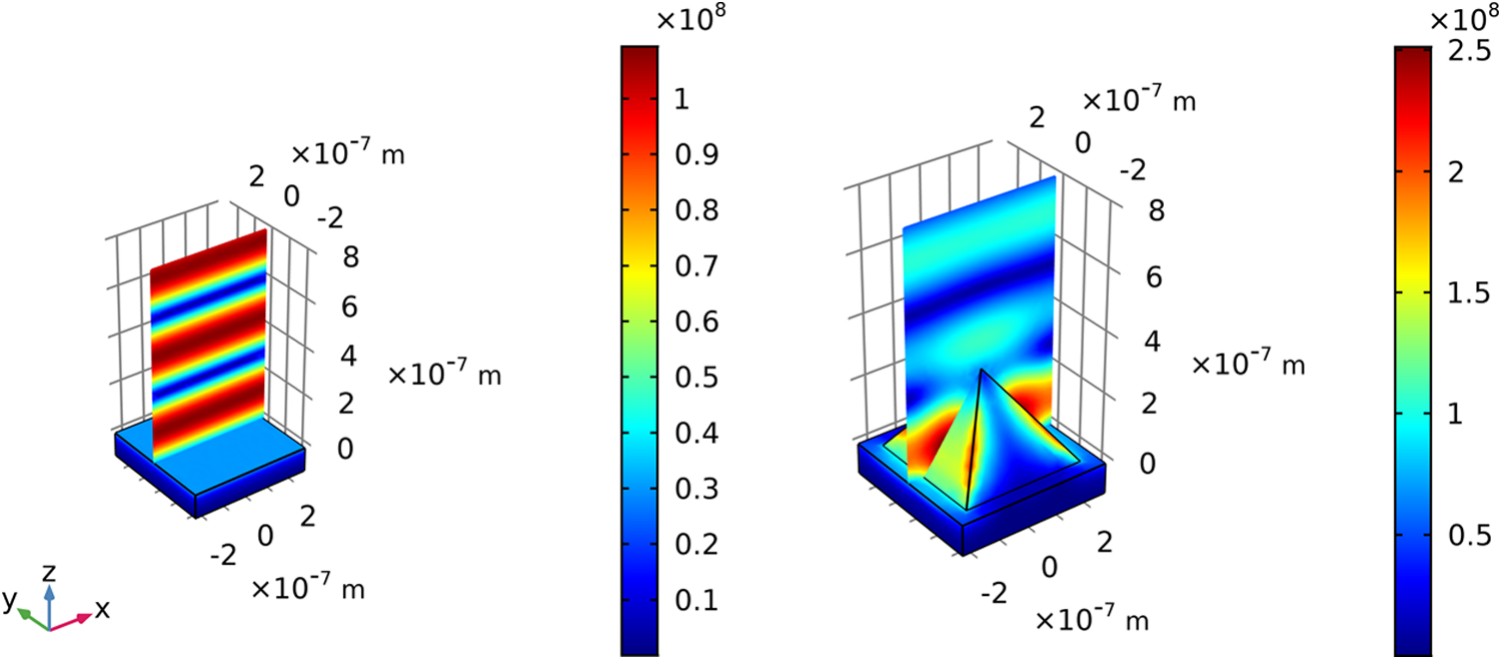
Distribution maps of the norm of the electric field (∥**E**∥) for a plane gold substrate (left) and for a gold substrate with pyramids (right). Incident plane wave with θ = 0° and ϕ = 0°, pyramid edge *L* = 400 nm, and spacing *D* = 100 nm.

The next parameter analyzed was the azimuth angle (ϕ) and its impact on the distribution of the electric field on the surface of the SERS substrate, as shown in Figure 3. Starting from ϕ = 0° — where the polarization is parallel to the *x* axis in Figure 3 - to ϕ = 45° - where **E** is aligned along the diagonal direction of the pyramid, the intensity of the electric field was plotted on the surface of the SERS substrate. Due to the symmetrical nature of the pyramidal structures, these two angles correspond to the maximum intensity of resonance in the optical response analyzed, as observed in our previous work.^43^ Based on this behavior, our SERS study was conducted specifically at these two angles to optimize signal enhancement.

**Figure 3:**
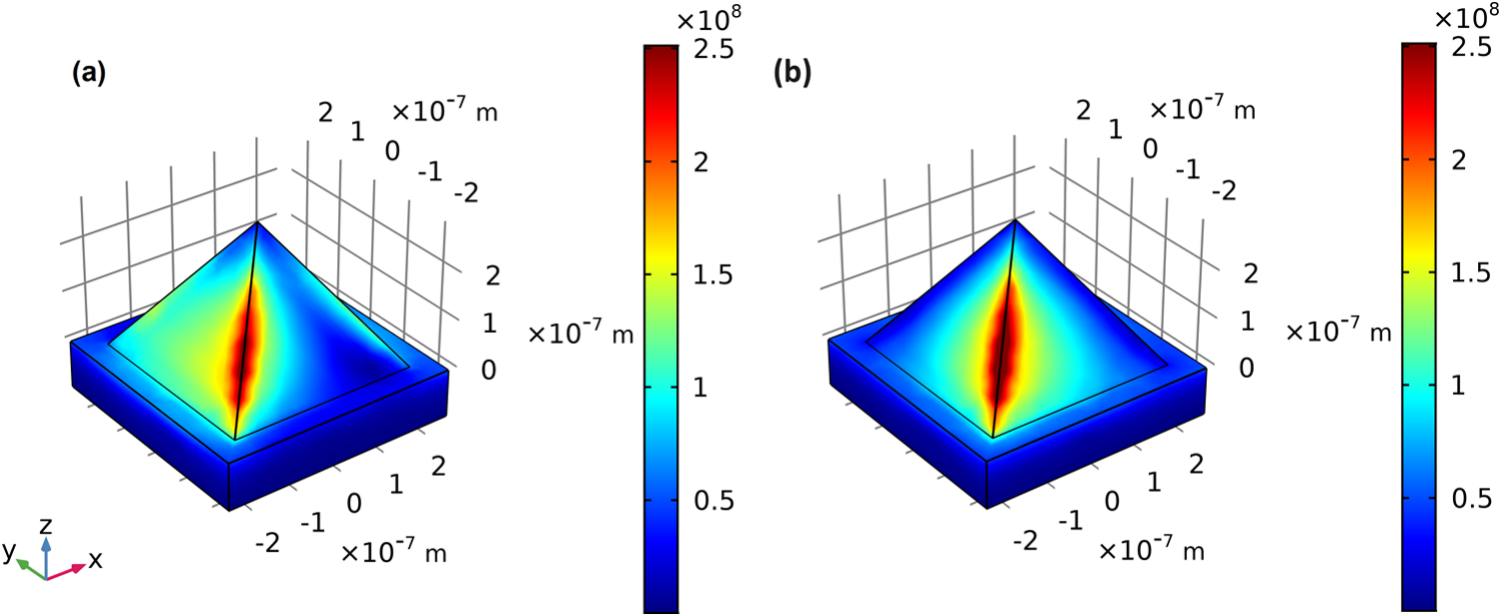
Distribution maps of the norm of the electric field (∥**E**∥) for a gold substrate with pyramids whose dimensions are *L* = 400 nm and *D* = 100 nm. Incident plane wave with θ = 0° and variable values of azimuth angle: (a) ϕ = 0°; (b) ϕ = 45°.

For lower values of ϕ, the electric field is distributed along the four lateral edges of the pyramid and as the azimuth angle increases, it becomes concentrated within the two lateral edges aligned with the incident wave polarization. Furthermore, as the azimuth angle increases, the field intensity also increases, reaching its peak for ϕ = 45°. From eq 1 one knows that the enhancement factor will be greater as the field intensity also becomes larger, and even though there are more aspects to be considered, Figure 3 suggests that better SERS performance would be achieved by a pyramidal substrate being excited by a 45° polarized source.

The last step is to vary the geometry of the SERS substrate by simulating different configurations of pairs of *D* and *L* values. The length of the edge of the pyramid was varied in the 50 nm ≤ *L* ≤ 450 nm interval, and the pyramid spacing was varied in the 0 ≤ *D* ≤ 500 nm interval. An additional parameter to be considered is also the azimuth angle ϕ of the excitation source. In this work, we present only results for ϕ = 0°. For each configuration, the adapted enhancement factor of eq 1 was evaluated, which results in the heat map in Figure 4. The map represents the EF′ for the different geometric configurations excited by a certain polarization of the incident light. In the map, the geometric locations of eight fabricated arrays are indicated. The discussion of the experiments and results obtained with those arrays will be presented in the Section on Raman Scattering Measurements.

**Figure 4:**
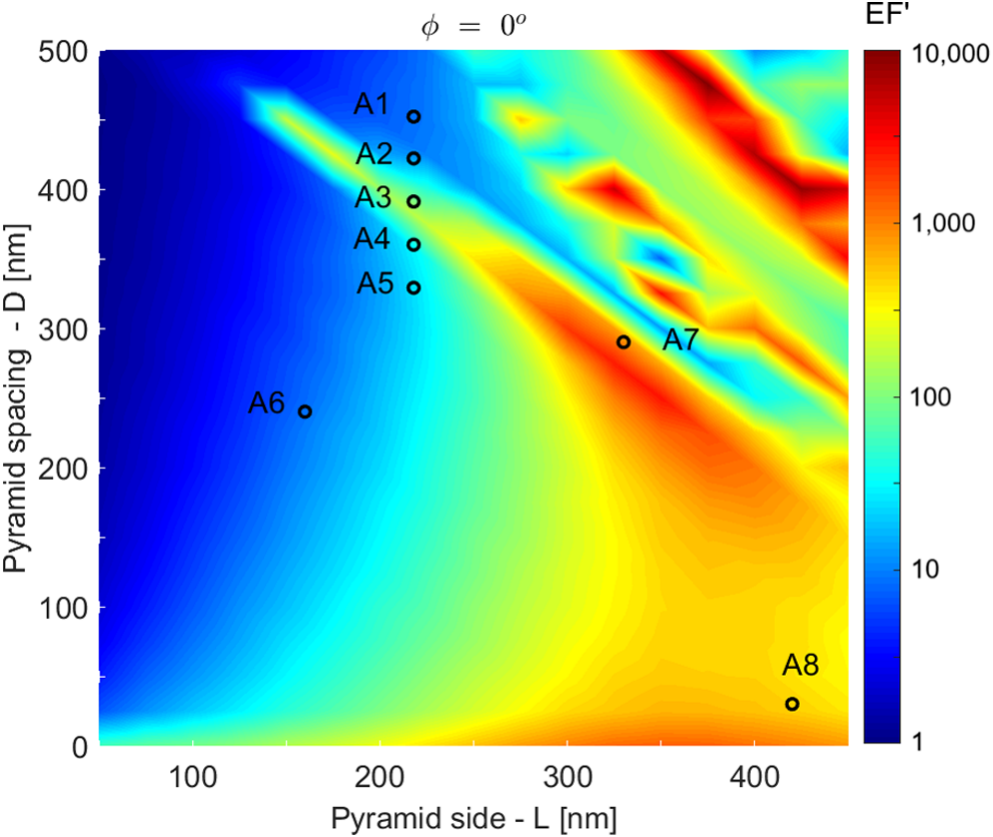
Adapted enhancement factor (EF′) as a function of pyramid edge length (L) and pyramid spacing (D) for ϕ = 0°. In the map, eight parameter pairs of fabricated arrays are indicated as A1 to A8. In Section 3.2, Raman Scattering Measurements, the results obtained with those arrays are presented.

As the heat map shows, depending on the chosen D,L configuration, the resulting EF′ can be very low, close to 1, which demonstrates the importance of the SERS substrate design process; there is a region that present significant EF′ for 300 nm ≤ *L* ≤ 400 nm and *D* close to 0 nm (EF′ ≈ 1000); there is a hot region aligned along the pitch *D* + *L* equal to 600 nm, and for 300 nm ≤ *L* ≤ 400 nm (EF′ ≈ 3000), and the hottest region in the map is located along a pitch ≈ equal to 850 nm and 350 nm ≤ *L* ≤ 450 nm (EF′ reaching ≈ 10,000). The peaks in EF′ occur in very narrow parameter regions.

In practice, our fabrication process is not yet perfect and the final structure is subject to small geometric fluctuations in pyramid size, spacing, and homogeneity.^44^ However, the pitch presents very small variations, in the nanometer range, due to the high precision of e-beam lithography in writing the pattern during template fabrication. In fact, the behavior observed in Figure 4 in the region close to (*D*,*L*) = (250 nm, 350 nm) shows that for the pyramid edge length between 300 and 400 nm, if the pitch can be maintained constant (*D* + *L* = 600 nm), the loss of EF′ due to fabrication imperfections is not critical. This characteristic translates into design robustness, which is an important feature in the context of SERS substrate fabrication.

Finally, a way to better understand the results in Figure 4 is to observe the behavior of the distribution of the norm of the electric field on the surface of the nanopyramid. Figure 5 presents such distributions for a pyramid with a hotspot geometry configuration (*D* = 250 nm, *L* = 350 nm) and for two azimuth angles, ϕ = 0° and ϕ = 45°. For ϕ = 0°, ∥**E**∥ is located on all four lateral edges of the pyramid, while for ϕ = 45°, most of the field is restricted to the two lateral edges that align with the polarization of the incident light. However, one can observe that the field intensity in the latter case is not only much higher, but also that this higher intensity extends over a larger area. From eq 1, EF′ depends both on the intensity of the electric field and the total area it covers. When comparing the two designs, it is now evident why 45° polarization yields a much better performance.

**Figure 5:**
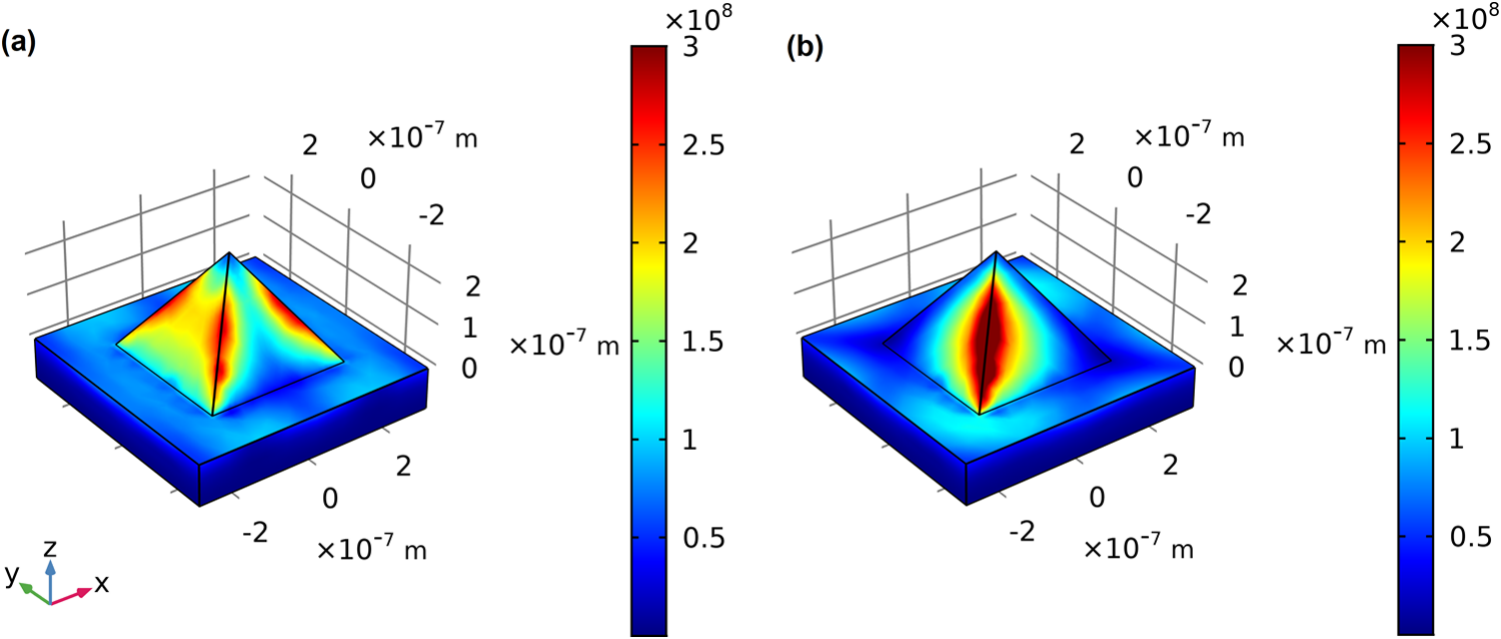
Distribution map of the norm of the electric field (∥**E**∥) on the pyramid surface of an array with dimensions *L* = 350 nm and *D* = 250 nm, corresponding to a hotspot in the map of Figure 4. (a) Normal incidence with ϕ = 0°. (b) Normal incidence with ϕ = 45°.

## Experimental Results and Discussion

Using the results presented in Figure 4, some pairs (*D*, *L*) were chosen to demonstrate the robustness of the simulations. Templates for arrays A1 to A5 were fabricated to demonstrate the presence of the structure aligned with the 600 nm pitch, A6 was chosen in a region with low enhancement, A7 was chosen next to a high hotspot on the map with *D* = 250 nm and *L* = 350 nm, and A8 was chosen to be near another hotspot with a large areal density of structures. The next step was then to fabricate the SERS substrates with the chosen dimensions. Afterward, Raman scattering measurements were performed with the fabricated substrates using urea as an analyte.

### Device Fabrication

The process of fabricating the SERS substrate involves several distinct steps. The first stage is to produce a template in silicon with an array of pyramidal cavities, following the steps presented in Figure 6.

**Figure 6:**
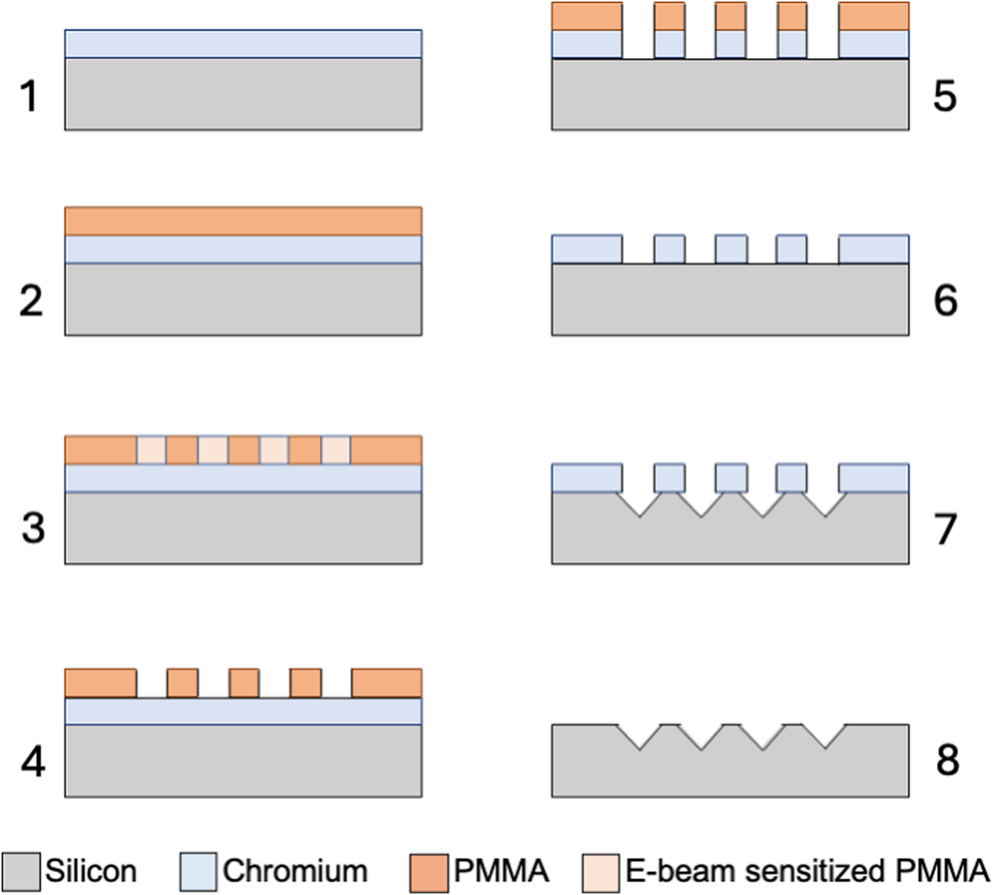
Fabrication steps for the template of pyramidal cavities. (1) (100) oriented silicon wafer covered with 40 nm thick chromium layer, (2) PMMA deposition, (3) Pattern definition on PMMA by e-beam lithography, (4) removal of the sensitized PMMA regions, (5) Chromium etching, (6) removal of remaining PMMA, (7) Silicon anisotropic etching with KOH solution, and (8) Chromium mask removal.

Step 1: The procedures for obtaining the array template start with a (100) silicon wafer being covered with a 40 nm thick chromium layer deposited by thermal evaporation. Step 2: A layer of poly­(methyl methacrylate) (PMMA) 950 K from Allresist GmbH, type AR-P 672.02, is spin-coated onto the sample at 8000 rpm, forming a 66 nm thick layer. A subsequent soft bake at 180 °C for 60 s evaporates the solvent, creating a uniform resist thin layer. Step 3: The patterning is generated by e-beam lithography, using a 30 kV Raith e-LiNe Plus electron beam lithography system. To generate a mask comprising a grid of circular windows with the appropriate diameter, a dose of 125 μC/cm^2^ is applied. Step 4: To improve development contrast, the sample undergoes cold development with a mixture of IPA and DI water (3:1 ratio) at −10 °C for 90 s. Step 5: The exposed chromium regions are then removed by immersing the sample in a chromium etching based on ceric ammonium nitrate 3Ce­(NH_4_)_2_(NO_3_)_6_ from MicroChemicals GmbH for 180 s at room temperature, exposing the silicon substrate beneath the circular apertures in the lithographic chromium mask. Step 6: Subsequently, the remaining PMMA layer is removed from the sample by immersion in acetone. Step 7: The sample is then immersed in a 40% potassium hydroxide (KOH) solution at 70 °C, resulting in the creation of a distinctive array of pyramidal-shaped cavities on the substrate surface. Step 8: The last step is to remove the remaining chromium mask with the same etching solution as in Step 5. The result of steps 1 to 8 of the sequence is a template in silicon of the desired array of pyramidal cavities, as shown in Figure 7.

**Figure 7:**
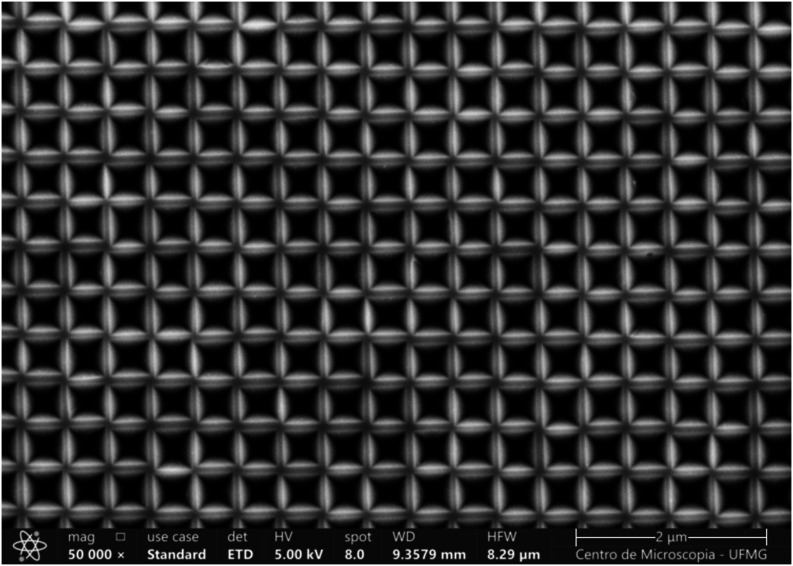
SEM image of an array template in silicon fabricated following the steps shown in Figure 6, with *L* = 0.42(2) μm and *D* = 0.03(2) μm.

The second stage is to fabricate the substrate using a template produced as described above and as shown in Figure 8. Step 1: Gold is thermally evaporated on the template. The thickness of the gold film is established as to be enough to fill the cavities. Step 2: A drop of epoxy resin is placed centered on top of the array. Step 3: The epoxy resin drop is pressed with a glass slide. The amount of resin is such as to cover only the array and a small border of the surrounding flat gold region. Step 4: After resin curing, the ensemble glass slide and conformal gold pyramids array are mechanically extracted, since gold and native oxidized silicon do not present good adhesion. Step 5: The final structure is a matrix of gold pyramids on a gold layer glued to a glass slide. Figure 9 shows a SEM image of a fabricated SERS substrate with average edge length *L* = 0.20(1) μm and spacing *D* = 0.25(1) μm. It is important to note that the silicon template remains intact and undamaged after the SERS substrate has been removed and can be reused. Thus, this fabrication process provides an efficient and inexpensive method for the large-scale production of SERS substrates.

**Figure 8:**
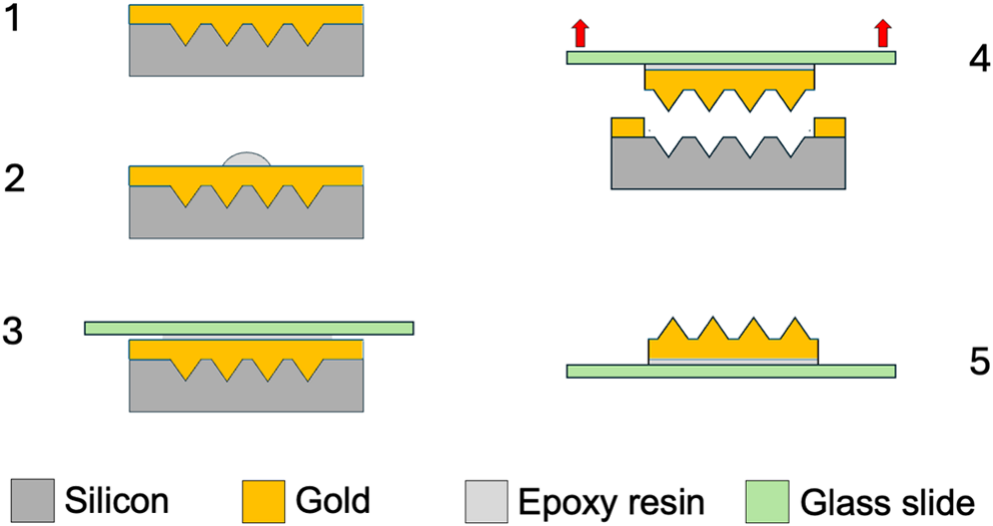
Fabrication steps of the pyramidal SERS substrate: (1) deposition of a gold layer by vacuum evaporation, (2) drop of the epoxy resin, (3) pressing of glass slide and curing of the epoxy resin, (4) extraction of the glass slide, and (5) SERS substrate.

**Figure 9:**
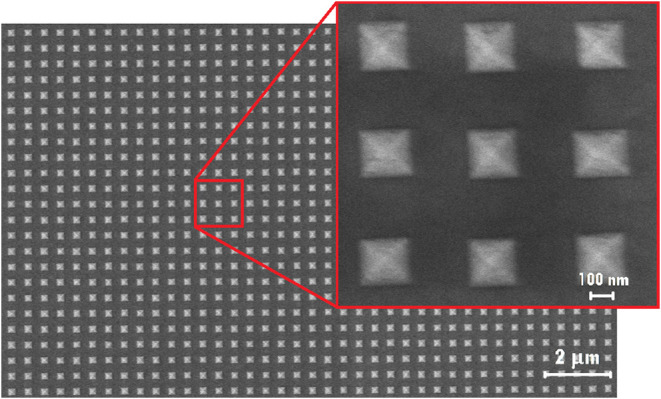
SEM image of a fabricated SERS substrate using an array of pyramidal cavities, comprising a matrix of square-based gold pyramids on a gold layer, with average pyramids edge length *L* = 0.20(1) μm and spacing *D* = 0.25(1) μm.

### Raman Scattering Measurements

To verify the design strategy and evaluate the performance of the fabricated SERS substrates, eight different array geometries were chosen, as indicated by the circles in Figure 4. Arrays A1 to A5 have the same nominal pyramid side *L* = 0.22(1) μm and varying distance between pyramids *D* (0.45(1) μm, 0.42(1) μm, 0.39(1) μm, and 0.33(1) μm, for A1 to A5 sequentially). This set of arrays was fabricated to confirm the results of the simulations that show a structure peaked approximately at the pitch (*L* + *D* = 600 nm) in that region of the map. Array A6 (*L* = 0.16(1) μm and *D* = 0.24(1) μm) was chosen to be in a cold region of the map. Arrays A7 (*L* = 0.33(1) μm and *D* = 0.29(1) μm) and A8 (*L* = 0.42(2) μm and *D* = 0.03(1) μm) are placed in hot regions.

The spectroscopic experiments were performed on a WiTec alpha 300 RA confocal Raman spectrometer using 633 nm laser light excitation. The target material chosen for the Raman scattering measurements was a diluted solution of urea, as it is nontoxic, nonflammable, and noncorrosive, and often used as a standard test sample in Raman spectroscopy.^45–47^ Pure ECIBRA urea 99% was used. Figure 10 shows the Raman spectrum of a thick layer of the urea powder used in our experiments pressed on a glass slide. All expected urea peaks, as reported in the literature, are present in the shown spectrum.^45–49^

**Figure 10:**
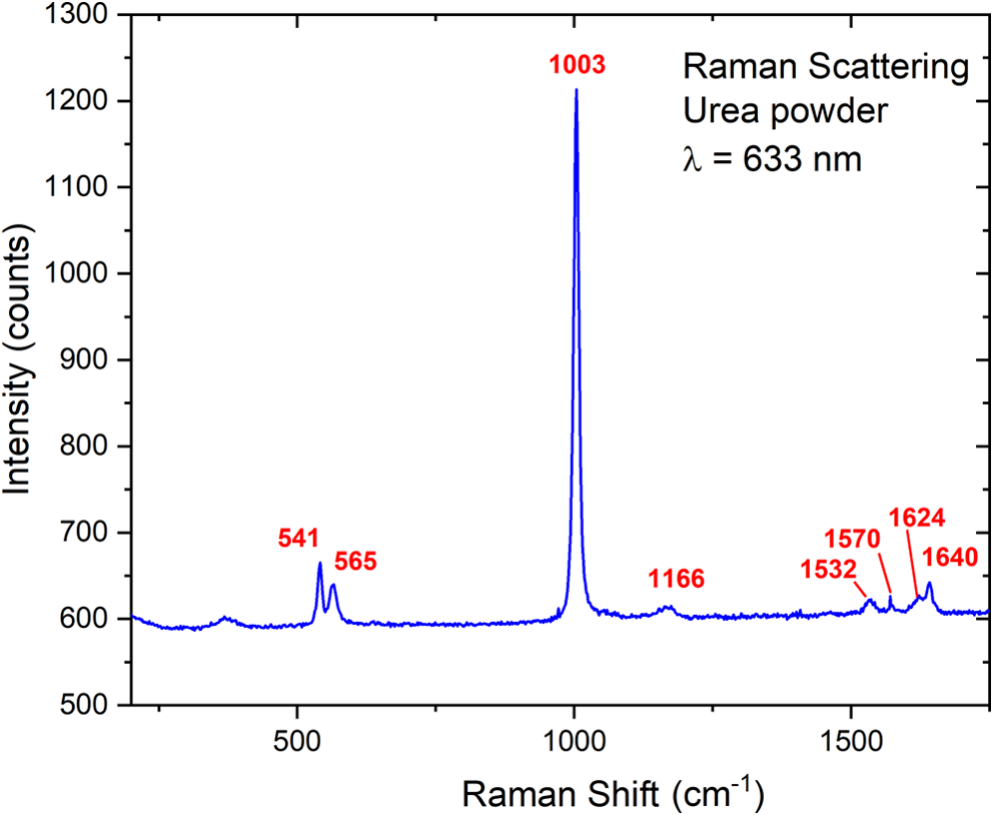
Raman spectrum of urea powder, with the main peaks indicated.

For each fabricated geometry, the aqueous urea solution, with a concentration of 40 mM/L, was deposited on the array of nanopyramids and also on the flat gold border that surrounds it. After the solution dried, the Raman signals from both locations were taken.

Figure 11 shows the image of substrate A7 after deposition and drying of the urea solution. As can be observed, the urea deposit presents some heterogeneity. The Raman measurement points are indicated by the cross-hairs, and they were chosen to avoid the darker deposit lines. The numerical aperture of the objective used in the Raman experiments was 0.55, which results in a theoretical value of 1.4 μm for the focal diameter.

**Figure 11:**
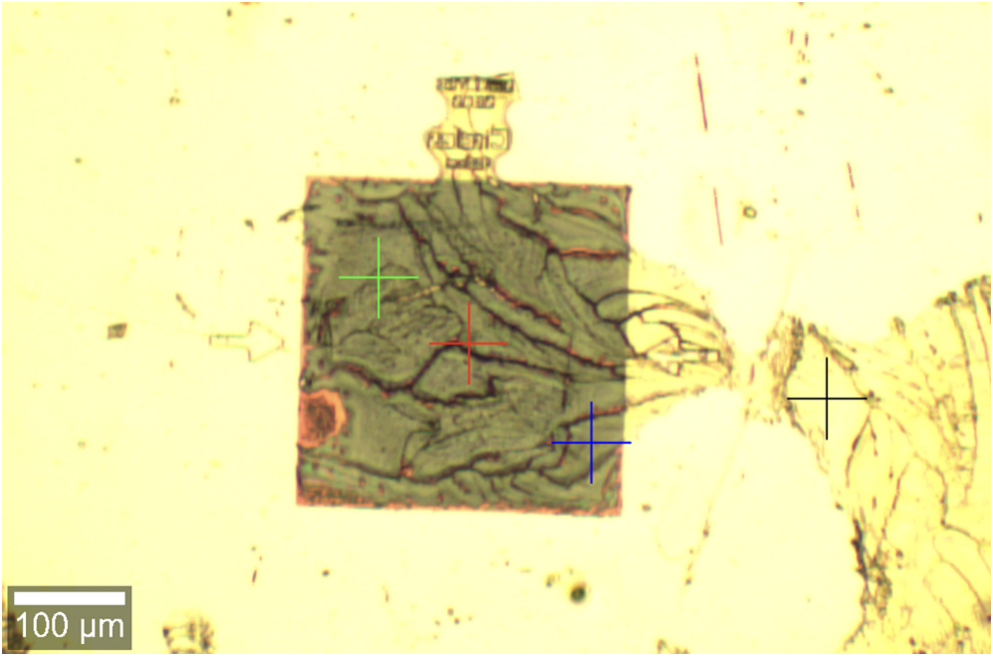
Optical image captured after the deposition and drying of the urea solution on substrate A7. The urea deposit showed some heterogeneity. The measurement positions were chosen to avoid the darker deposit lines. The 300 μm × 300 μm A7 array was measured at three positions, indicated by the green, red, and blue cross-points. The flat gold was measured at the black cross-point.

The main Raman peak of urea is the feature around 1000 cm^–1^.^45–49^ In Figure 12 the main Raman urea peaks for the three positions in array A7 are shown. The heterogeneity of the urea deposit is reflected in the lower counts obtained at the red cross-point. The enhancement *E* observed for each position in the array is obtained from the ratio of the peak height for the urea peak around 1000 cm^–1^ at the position and the peak in the flat gold region: *E* = 15.50 for the green cross-point; *E* = 9.89 for the red cross-point and *E* = 15.89 for the blue cross-point.

**Figure 12:**
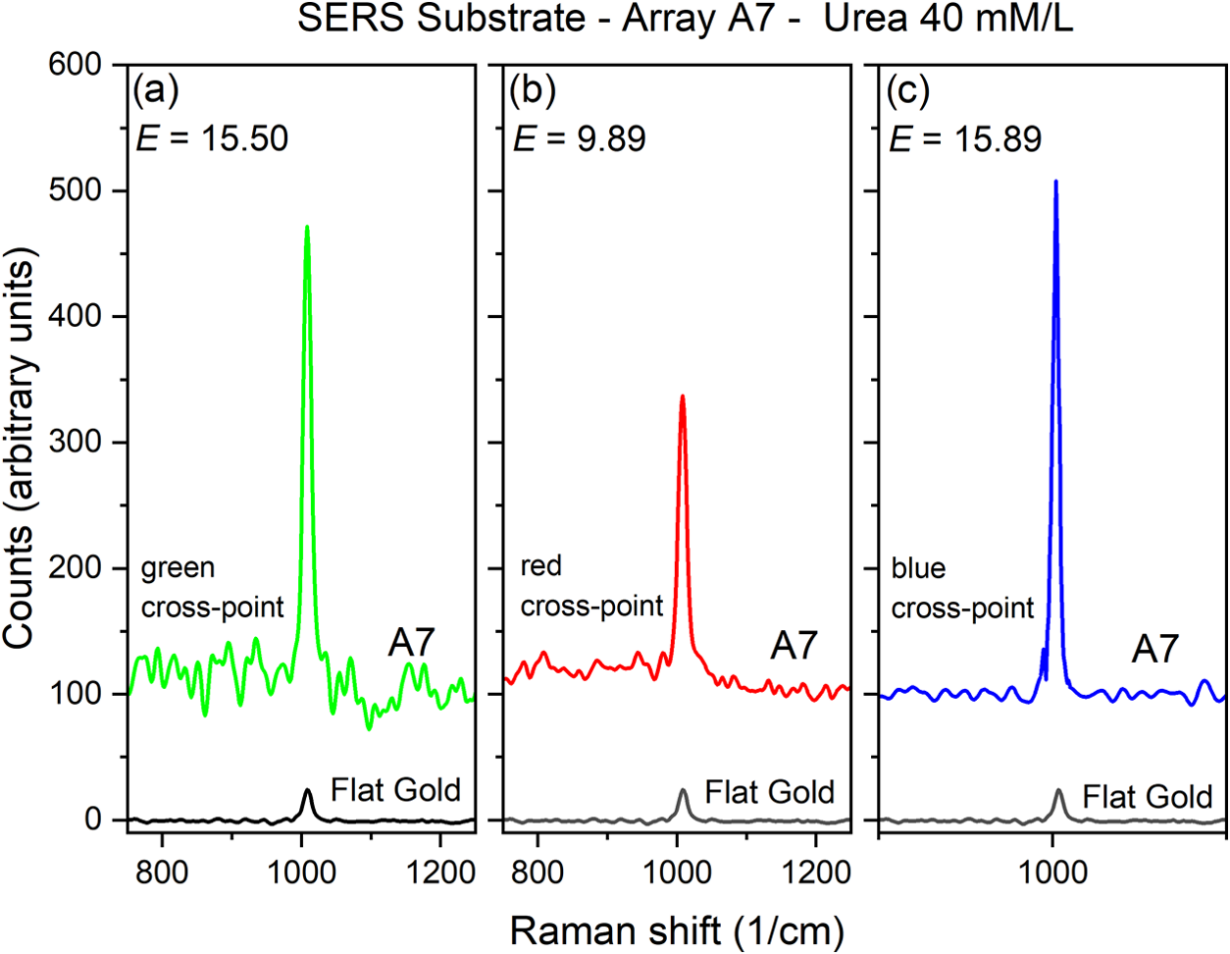
Raman spectra of urea 40 mM/L at three positions on array A7 and also on the flat gold region around the array, as indicated in Figure 11.

In Figure 13, the Raman spectra for the 40 mM/L urea aqueous solution applied on the A1 to A5 arrays and for the flat gold region around them are shown. As expected, the intensity of the Raman urea spectra scales with the local values of EF′ as indicated by Figure 4. As the sequence crosses the feature indicated on the map, the largest Raman signal intensity occurs for A3, since its dimensions locate it near a hotter region in the map than A1, A2, A4, and A5.

**Figure 13:**
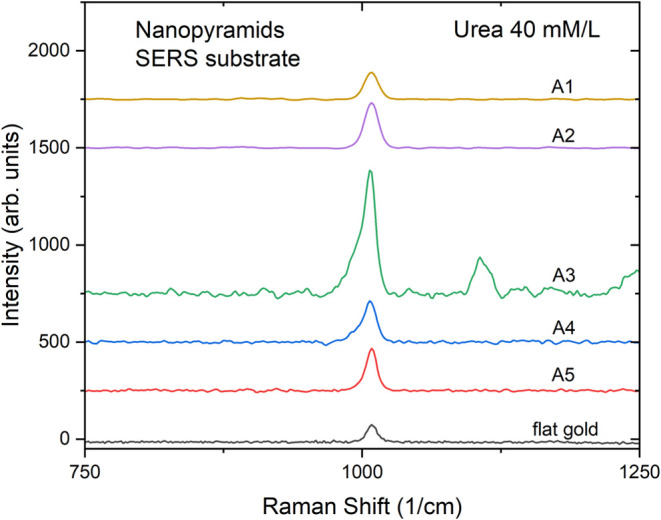
Raman spectra of urea 40 mM/L on the arrays A1 to A5, as indicated in Figure 4, and also on the flat gold region around the arrays.

The comparison of the results for the arrays A6, A7, and A8 is shown in Figure 14.

**Figure 14:**
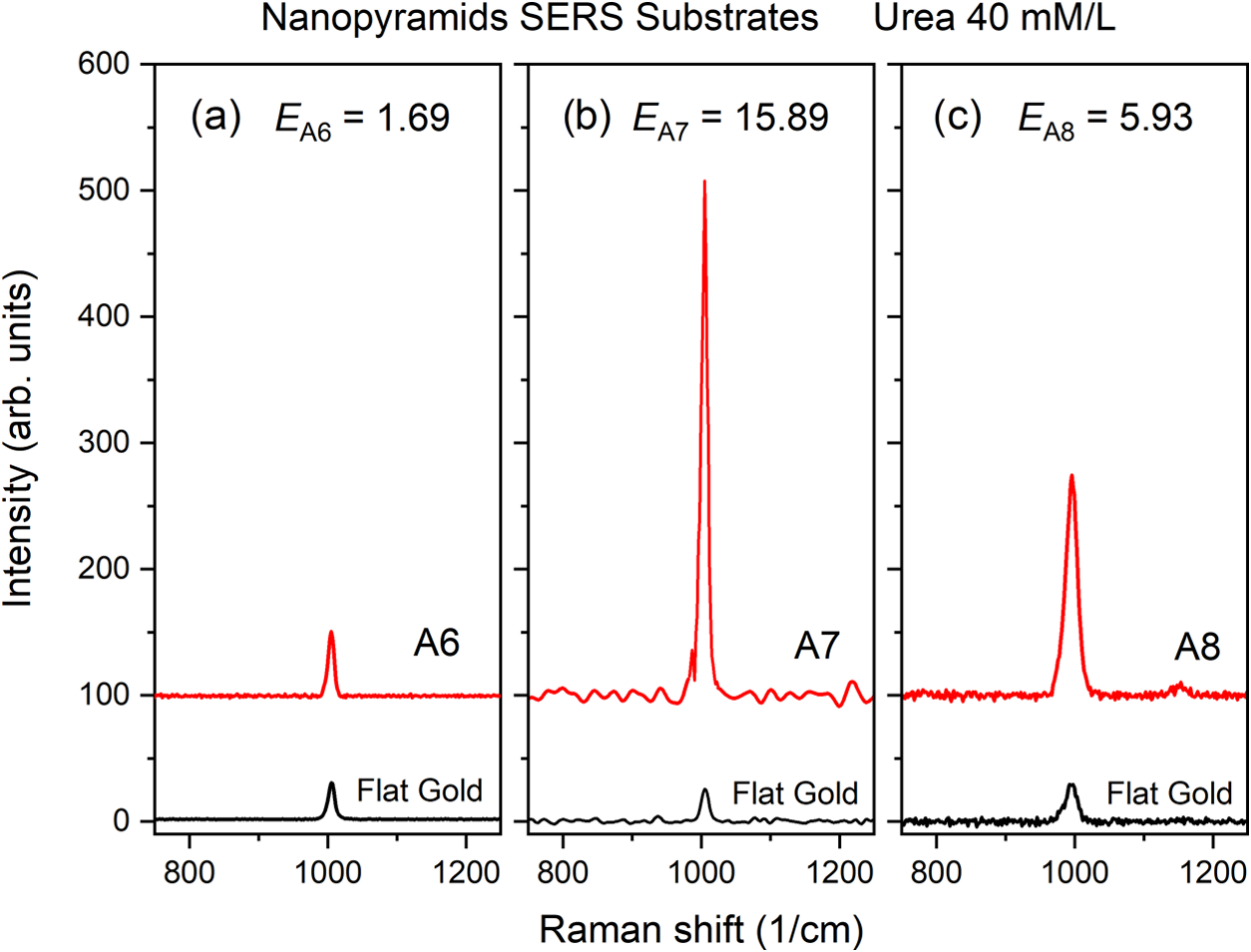
Raman spectra of urea 40 mM/L on (a) array A6, (b) array A7, and (c) array A8, and respective flat gold spectra. The experimental enhancement *E* for each array is indicated.

The enhancement *E* for each array was obtained as described above: *E*_A6_ = 1.69; *E*_A7_ = 15.89 and *E*_A8_ = 5.93. These values corroborate the trends expected by the simulations.

## Conclusions

This work presents the proposal, design, and experimental demonstration of a structured SERS substrate comprising a matrix of uniformly distributed square gold pyramids on a gold film. We employ 3D-FEM techniques for the design phase, optimizing the geometrical parameters of the pyramids (edge length and spacing) and the incident light polarization characteristics.

Our numerical simulations provided crucial information on the behavior of the electromagnetic field on the substrate surface. The optimized structure achieved an enhancement factor (EF) of approximately 10^8^ relative to a flat gold substrate.

Our fabrication process utilizes a reusable silicon mold for the production of SERS substrates. This approach enables high throughput while maintaining excellent device reproducibility.

Raman spectroscopy measurements using 40 mM/L urea demonstrated approximately 1500% enhancement with our engineered substrates compared to flat gold substrates. These results validate our design methodology and, combined with our novel production strategy, present a promising approach that enables the serial production of high-quality SERS substrates.

## ⊥

Materials Research Laboratory, University of Illinois, Urbana, Illinois 61801, United States
